# Targeting TB or MRSA in Norwegian municipalities during ‘the refugee crisis’ of 2015: a framework for priority setting in screening

**DOI:** 10.2807/1560-7917.ES.2019.24.38.1800676

**Published:** 2019-09-19

**Authors:** Anders Skyrud Danielsen, Petter Elstrøm, Trude Margrete Arnesen, Unni Gopinathan, Oliver Kacelnik

**Affiliations:** 1 Department of Antibiotic Resistance and Infection Prevention, Norwegian Institute of Public Health, Oslo, Norway; 2 Department of Tuberculosis, Blood Borne and Sexually Transmitted Infections, Norwegian Institute of Public Health, Oslo, Norway; 3 Cluster for Global Health, Norwegian Institute of Public Health & Institute of Health and Society, University of Oslo, Oslo, Norway

**Keywords:** tuberculosis, MRSA, refugees, screening, register, policy evaluation, migrants, asylum seekers

## Abstract

**Introduction:**

In 2015, there was an increase in the number of asylum seekers arriving in Europe. Like in other countries, deciding screening priorities for tuberculosis (TB) and meticillin-resistant *Staphylococcus aureus* (MRSA) was a challenge. At least five of 428 municipalities chose to screen asylum seekers for MRSA before TB; the Norwegian Institute for Public Health advised against this.

**Aim:**

To evaluate the MRSA/TB screening results from 2014 to 2016 and create a generalised framework for screening prioritisation in Norway through simulation modelling.

**Methods:**

This is a register-based cohort study of asylum seekers using data from the Norwegian Surveillance System for Communicable Diseases from 2014 to 2016. We used survey data from municipalities that screened all asylum seekers for MRSA and denominator data from the Directorate of Immigration. A comparative risk assessment model was built to investigate the outcomes of prioritising between TB and MRSA in screening regimes.

**Results:**

Of 46,090 asylum seekers, 137 (0.30%) were diagnosed with active TB (notification rate: 300/100,000 person-years). In the municipalities that screened all asylum seekers for MRSA, 13 of 1,768 (0.74%) were found to be infected with MRSA. The model estimated that screening for MRSA would prevent eight MRSA infections while prioritising TB screening would prevent 24 cases of active TB and one death.

**Conclusion:**

Our findings support the decision to advise against screening for MRSA before TB among newly arrived asylum seekers. The model was an effective tool for comparing screening priorities and can be applied to other scenarios in other countries.

## Introduction

Tuberculosis (TB) is the leading cause of death from infectious diseases worldwide [1] and has been considered a global health problem for over a century. The World Health Organization estimates that over 10 million people fall ill with TB annually and over 1.5 million die from the disease [2]; it is estimated that up to 25% of the global population has a latent TB infection [3]. Meticillin-resistant *Staphylococcus* *aureus* (MRSA) are strains of the *S.* *aureus* bacterium that are resistant to several antimicrobials and, like the sensitive strains, can colonise the skin of humans. Both resistant and sensitive *S. aureus* can also cause invasive infections. Methicillin-resistance in *S. aureus* is found at very different rates globally, from almost half of all clinical isolates of *S. aureus* in some European countries to less than 1 % in northern Europe [4]. In non-hospitalised people, *S. aureus* seldom causes severe infections, however, it is one of the most common pathogens causing severe nosocomial infections. The number of deaths attributable to MRSA has increased by 28% in Europe from 2007 to 2015 [5].

Both TB and MRSA are important public health problems globally. In Norway, the detections of TB and MRSA notified to the national surveillance systems are low. The yearly notification rate of TB in Norway was six per 100,000 population in 2016 [6]. The prevalence of latent tuberculosis infection (LTBI) in Norway is not known. The notification rate of MRSA was 49 per 100,000 person-years (PY) in 2016, of which 35% were clinical infections [7]. In the same year, 1% of *S. aureus* isolates from blood cultures were MRSA [7]. In comparison, the proportion of strains resistant to meticillin among clinical *S. aureus* isolates from Russia was 66.5% [4] and 80 per 100,000 population for TB (of which, 42/100,000 population were multidrug-resistant or rifampicin-resistant) [8]. Contact tracing in hospitals has shown carriage of MRSA in 0.31% of healthcare personnel in Norway, demonstrating a low prevalence of carriage in the population [9].

An important tool in the prevention and control of TB is surveillance and detection through targeted screening. Previous studies have found an increased risk of infectious disease transmission among asylum seekers, specifically for TB [10,11]. All asylum seekers entering Norway should be screened for TB within 2 weeks of arrival, although rare exceptions do occur, e.g. if the asylum seeker is moved to another location, thereby delaying the test. This TB screening programme is mandatory in Norway [12]. Screening consists of a pulmonary X-ray for persons aged 15 years or older in addition to interferon gamma release assay (IGRA) testing for persons aged 35 years or less; positive findings are further investigated by an infectious disease specialist. All persons with active TB are treated, while those who are suspected of having a latent infection, based on an algorithm provided by the Norwegian Institute of Public Health (NIPH), are considered for voluntary preventive treatment.

MRSA screening is only recommended if the person encountering Norwegian healthcare institutions meets certain criteria, for example, that they have spent time in a refugee camp [13]. Patients are screened by taking swabs from skin or mucous membranes before or on admission to hospitals [14].

During 2015, there was an increase in the number of people seeking asylum in European countries, with several countries facing tough choices regarding prioritising resources within healthcare [15,16]. Previous studies have indicated that European countries vary in how they organise screening practices targeting asylum seekers and that these programmes likely faced resource constraints [17,18]. In Norway, 31,150 individuals applied for asylum, almost three times as many as in 2014 (11,480) and almost 10 times as many as in 2016 (3,460) [19]. In response to the sudden increase, the NIPH made a temporary adjustment of the screening programme in November 2015, prioritising screening for active pulmonary TB and partly omitting and postponing screening for LTBI [20]. During this period, asylum seekers often had to move from municipality to municipality during their initial transit stay, creating a disorganised situation where asylum seekers could be moved before the screening results were available [21].

Some asylum seekers meeting certain criteria should undergo MRSA screening before non-acute contact with hospitals [13]. Determining who meets the criteria can be difficult due to language and cultural barriers. To prevent the spread of MRSA, municipal medical officers in at least five of the 428 Norwegian municipalities introduced screening for MRSA at their respective district hospitals for all asylum seekers before TB screening. The resulting delay in TB screening, as MRSA status needed to be confirmed first, meant that some asylum seekers were moved to another asylum centre before a TB test could be performed at all. The NIPH advised against this practice, underlining the greater importance of quickly clarifying their TB status rather than diverting resources to MRSA screening, because of TB’s epidemic potential [22]. In addition to this, the TB screening would likely be the only interaction a healthy asylum seeker would have with Norwegian healthcare. As such, the only medical indication for an MRSA test would be to provide special infection control measures at the TB screening station, unless the plan was to sanitise the MRSA infected asylum seeker, a procedure that is not recommended for a healthy carrier of MRSA in Norway.

In this study, we evaluated whether the advice not to screen for MRSA before screening for TB among newly arrived asylum seekers entering Norway in 2015 was reasonable and whether this practice increased the risk of TB and MRSA transmission. To investigate this, we found the occurrences of both MRSA and TB among asylum seekers in Norway from 2014 to 2016. Then, we modelled the effect that different screening options would have on disease transmission, morbidity and mortality as a consequence of undetected cases. The framework we built can guide similar prioritisations in the future, as the control and prevention of different infectious diseases in Europe may become a pressing issue in a more globalised world where healthcare resources are scarce.

## Methods

### Study design

Our study was a register-based cohort study using data on newly arrived asylum seekers from the Norwegian Surveillance System for Communicable Diseases (MSIS) during 2014, 2015 and 2016. The study was carried out in two parts, where we first described the occurrences in the cohort, before modelling different scenarios.

### Data sources

#### Norwegian Surveillance System for Communicable Diseases

Doctors and laboratories are required by law to notify cases for 71 notifiable diseases in Norway, including MRSA and TB [23]. Reporting clinicians and local and reference laboratories also provide epidemiological data (e.g. place of residence, time of diagnosis, age of the patient) and microbiological data (e.g. genetic strain of the microbe, resistance) [24]. For this study, data from the TB and MRSA registers were linked using the name and date of birth date for each individual.

#### Directorate of Immigration

Data on the number of asylum seekers entering Norway in 2014, 2015 and 2016 and their countries of origin were collected from the Directorate of Immigration (UDI) and used as denominators for the analyses [19]. This data was not linked on an individual level.

### Participants

The study population comprised of all newly arrived asylum seekers entering Norway from 2014 to 2016, a total of 46,090 individuals.

To identify the screening routines implemented in 2015 (i.e. screening for MRSA or TB first), a survey was sent to municipal medical officers in 34 municipalities in Norway (those with asylum centres). Sixteen municipalities answered the survey, of which five reported having tested all asylum seekers for MRSA before testing for TB. The local denominator (the number of asylum seekers registered in the respective municipality) was collected from the statistics department of the UDI [19].

### Modelling the effect of different screening regimes

We built a data-driven, probabilistic and untimed comparative risk assessment model with Markovian properties that was run as a Monte Carlo simulation with 1,000 iterations [25]. The model used probabilities and empirical values from the cohort study and values found in the literature as parameters and disease outcomes as outputs. Outcomes from two screening regimes were compared in the model — one for MRSA screening before TB screening and one where TB screening was prioritised with no MRSA screening, assuming that if the TB screening is done before the MRSA screening, there is no longer any medical indication for an MRSA test. The model only includes asylum seekers living in asylum centres and transmission within this group, as hospitalised asylum seekers are screened for MRSA on admission and were therefore removed from the model [13].

An assumption in the model is that only one of the screening strategies can be implemented at a given time (this assumption will be discussed in more detail later). We also assume that adding a screening programme would reduce the transmission reflecting an 85% sensitivity for the respective diseases, based on conservative estimates of the mean screening sensitivity found in the literature [26,27].

**Figure 1:**
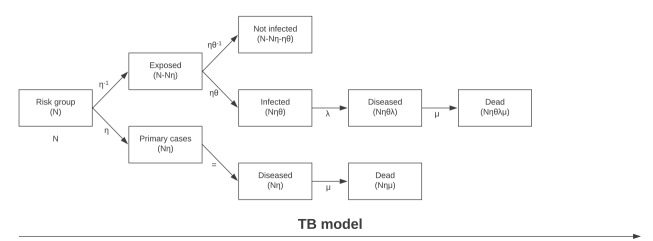
Model scenario for tuberculosis screening, Norway, 2014–2016

**Figure 2:**
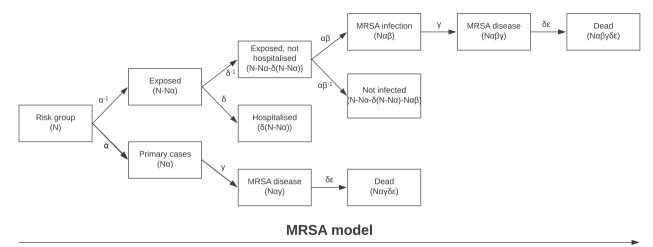
Model scenario for MRSA screening, Norway, 2014–2016^a^

The model was constructed and run in Microsoft Excel 2016. Diagrams of the model scenarios can be seen in Figure 1 and Figure 2 and the parameters included in the model are described in Table 1. As the outcomes could be seen as discrete counts, the beta distribution were chosen due to its relationship and approximation to the negative binomial distribution for large samples.

MRSA: meticillin-resistant *Staphylococcus aureus*.

^a^ The MRSA model uses the estimate from the provisory 2015 screening.

**Table 1:** Parameters included in the model to estimate the effect of different screening regimes, Norway, 2014–2016

Variables in the model		Distribution	Estimate (uncertainty interval)	Notes
**MRSA parameters**				
N	Asylum seekers	NA	46,090	Number of asylum seekers in the study period [19]
α	Probability of MRSA in newly arrived asylum seekers	Beta	0.0074 (0.00342–0.01138)	Estimate of MRSA among asylum seekers as reported in the results section
β	MRSA basic reproduction number	NA	1	Estimate based on [36] and [30]
δ	Probability of hospitalisation	Beta	0.16 (0.14–0.18)	Estimated probability based on statistics from Statistics Norway [37]
γ	Probability of MRSA progression to infection	Beta	0.035 (0.0008–0.11)	Estimated probability from published literature [36,38,39]
τ	Probability of MRSA bacteraemia in MRSA positive inpatients	Beta	0.02 (0.01–0.03)	Estimate based on MSIS register data and [40]
ε	Mortality for MRSA bacteraemia inpatients	Beta	0.223 (0.107–0.474)	Estimate from [41]
ζ	Mortality for MSSA bacteraemia inpatients	Beta	0.202 (0.173–0.221)	Estimate from [41]
**TB parameters**				
N	Asylum seekers (risk group)	NA	46,090	Number of asylum seekers in the study period [19]
η	Probability of active TB disease	Beta	0.003 (0.001–0.005)	Estimate of TB as reported in the results section
θ	TB basic reproduction number	NA	2	Estimate based on [42] and [43]
λ	Progression from latent to active TB	Beta	0.10 (0.025–0.175)	Lifetime risk of progression, from [44]
μ	Mortality for TB among active TB cases	Beta	0.035 (0.02–0.05)	Mortality (including those in treatment) for western countries from [28]

MRSA: meticillin-resistant *Staphylococcus aureus*; MSIS: Norwegian Surveillance System for Communicable Diseases; MSSA: methicillin-susceptible *S. aureus*; NA: not applicable; TB: tuberculosis.

The output of the model is the morbidity and mortality resulting from the transmission of the diseases as a consequence of undetected cases during screening, or because of lack of or delayed screening. The equations behind the outcomes measured can be found in Supplementary Table S1. The outcomes are assessed by calculating the mean of the iterations in the simulation and using the 2.5th percentile and 97.5th percentile as uncertainty intervals.

For MRSA, the primary cases in the model are the estimated number of asylum seekers entering Norway with MRSA infection, carriage or colonisation during 2014, 2015 and 2016, using the estimate from the notified cases from the MRSA screening municipalities in 2015. For TB, a primary case was an asylum seeker with active TB disease upon arrival estimated from the number of asylum seekers registered in MSIS with active TB disease within 3 months of arrival. Both MRSA and TB have latent or asymptomatic conditions where the pathogenic agent has colonised the host without causing disease. Secondary cases are all persons infected by the primary cases, including both persons with latent infections or colonisation and persons with active infection.

In the model, the number of secondary cases is the product of the primary cases multiplied by the basic reproduction number. The primary and secondary cases of the respective disease are added together and multiplied by the rate applied for the probability of progressing from the latent condition to active TB disease or MRSA infection. MRSA infections are defined as all infections, ranging from skin and soft tissue infections to fatal bacteraemia. Only those with severe MRSA infections (i.e. bacteraemia and endocarditis) contribute to the mortality rate. The mortality for these infections is then calculated. For TB infection there is good data on the number of patients that die in each country or region of the world. This includes patients receiving treatment [28]. For MRSA infections, however, a common cause of death is severe infection among hospitalised patients. The probability of death associated with MRSA is calculated by multiplying the total number of MRSA cases with the probability of hospitalisation together with the probability for bacteraemia among inpatients and the mortality among inpatients with bacteraemia (Supplementary Table S1). This gives the number of primary and secondary cases expected to die from *S. aureus* bacteraemia. To calculate the added risk of death attributable to the resistance mechanism itself, we subtracted the probability of dying from meticillin-sensitive *S. aureus* (MSSA) from the probability of dying from MRSA.

To simulate a screening situation, the reproduction of the respective diseases is dampened with the screening sensitivity factor mentioned earlier, corresponding to (N*αβ *− *δ*))0.85 for MRSA and (*θ*N*η*)0.85 ηfor TB.

### Sensitivity analyses

Since the basic reproduction number for TB and MRSA was assumed to be the uncertain parameter with the biggest impact on outcomes, it was chosen as the target for sensitivity analysis. The sensitivity analysis was performed by estimating the minimum and maximum results when changing the values for the reproduction number. The reproduction rate ranged from 0.5 to 3.5 for the TB analysis and 0.5–1.5 for MRSA. These analyses can be found in Supplementary Figure S1.

### Ethical statement

The study protocol was approved by the Regional Ethics Committee of South-Eastern Norway (2017/1284) and authorised by the Norwegian Data Protection Authority (17/12717). The application for data was approved by the MSIS register with the basis in the MSIS regulation [23].

## Results

Between 2014 and 2016, there were 46,090 newly arrived asylum seekers, 34,667 (75.22%) were male and 32,380 (70.25%) were over the age of 18. The three most common countries of origin were Syria (28.15%; 12,976/46,090), Afghanistan (17.25%; 7,952/46,090) and Eritrea (13.91%; 6,410/46,090).

### Disease occurrence

#### Tuberculosis

Of 46,090 newly arrived asylum seekers, 137 were diagnosed with active TB disease within 3 months of arrival during the study period. This corresponded to a notification rate of 300 per 100,000 PY or 0.30%. 116 of the 137 TB patients were male (84.67 %) and the mean age was 23.66 years old (range 1–61 years). There are no statistical margins of error calculated for these rates, as these are values for the entire population of asylum seekers in Norway over the study period, although there could be errors in detection.

The 137 TB cases originated from a diverse group of countries with different endemic levels of TB (Table 2). The most cases of TB were observed among asylum seekers from Somalia, Eritrea and Sudan (Sudan and South Sudan were registered together during these years), all countries with a high TB incidence rate. The TB prevalence observed among the asylum seekers was higher than that of their respective country of origin (Table 2).

**Table 2:** TB prevalence found among asylum seekers by country of origin and incidence in the respective country of origin in 2015^a^, Norway, 2014–2016

Country of origin	TB prevalence by country of origin (%)	TB incidence rate/100,000 in the respective country of origin
Somalia	1.80	274
Eritrea	0.59	65
Sudan	0.57	88
Ethiopia	0.49	192
Afghanistan	0.36	189
Syria	0.10	20
Iraq	0.00	43
Iran	0.00	16

TB: tuberculosis*.*

^a^ As estimated by the World Health Organization [35].

#### Meticillin-resistant *Staphylococcus aureus*

In the five municipalities, where all asylum seekers were screened for MRSA upon arrival in 2015, the estimated prevalence was 0.74% (estimated 95% confidence interval (CI): 0.34–1.14) (Table 3). All but one of these patients were from Syria. The notification rate for all newly arrived asylum seekers from 2014 to 2016 was 0.56% (259/46,090) including those possibly screened upon arrival. Of the MRSA patients among all newly arrived asylum seekers, 62.16% (161/259) were male and the mean age was 20.71 years old (range 0–55). Between 2014 and 2016, of 259 notified MRSA cases among newly arrived asylum seekers, 23.17% (60/259) were positive for Panton-Valentine leucocidin (PVL). A comparison of MRSA cases in the general Norwegian population during 2014–2016, registered in the MSIS register, showed a proportion of 35.5% (2,192/6,175) PVL positive.

**Table 3:** Prevalence of MRSA from five municipalities that screened all asylum seekers for MRSA, Norway, 2015 (n = 13)

Municipality	Asylum seekers in 2015	MRSA cases in 2015	Detected MRSA (%)
A	173	1	0.58
B	437	1	0.23
C	407	2	0.49
D	362	4	1.10
E	389	5	1.29
**Total**	**1,768**	**13**	**0.74**

MRSA: meticillin-resistant *Staphylococcus aureus.*

### Modelling screening scenarios

We found that prioritising MRSA screening would reduce MRSA morbidity by eight infections in the modelled risk group. Our model predicts that TB screening would reduce TB morbidity by 24 cases of active TB and reduce the TB mortality by one death in the study group.

Table 4 shows the results of the simulation of the comparative risk assessment model. Only one screening programme was in effect at a time, identifying 85% of the primary cases and thus allowing 15% of the primary cases to spread the disease. The disease that in the actual strategy were not screened for, was permitted to spread freely but was detected and treated as it progressed from latent to active disease.

**Table 4:** Outcomes from the models with screening either for MRSA first or solely for TB, Norway, 2014–2016

Outcomes from screening	Estimated value (95% CI)	Minimum value (sensitivity analysis)	Maximum value (sensitivity analysis)
**MRSA screening prioritised**			
MRSA secondary cases	43 (23–67)	21 (12–33)	64 (37–100)
MRSA total infections	14 (1–65)	13 (0–58)	14 (1–67)
MRSA total mortality	0 (0–1)	0 (0–1)	0 (0–2)
Mortality attributable to meticillin resistance in *S. aureus*	0 (0–0)	0 (0–0)	0 (0–0)
Secondary LTBI	277 (126–487)	68 (31–116)	476 (219–855)
Total TB disease	28 (8–135)	7 (4–64)	48 (11–208)
Total TB mortality	6 (2–20)	5 (1–15)	6 (2–23)
**TB screening prioritised**			
MRSA secondary cases	288 (159–445)	142 (78–217)	430 (247–668)
MRSA total infections	22 (1–107)	17 (1–77)	27 (1–126)
MRSA total mortality	0 (0–2)	0 (0–2)	1 (0–3)
Mortality attributable to meticillin resistance in *S. aureus*	0 (0–0)	0 (0–0)	0 (0–1)
Secondary LTBI	41 (19–73)	10 (5–17)	71 (33–128)
Total TB disease	4 (1–13)	1 (0–3)	7 (1–24)
Total TB mortality	5 (1–13)	5 (1–12)	5 (1–14)

CI: confidence interval; LTBI: latent tuberculosis infection; MRSA: meticillin-resistant *Staphylococcus aureus*; TB: tuberculosis*.*

## Discussion

Through a comparative risk assessment simulation model, we found that there was a higher transmission (i.e. secondary cases) of TB when prioritising MRSA. Although the CIs were wide and partially overlapping, the point estimates showed additional TB disease and one additional death with delayed TB screening because of MRSA screening. While the MRSA screening could have reduced the number of MRSA infections, our model estimated no deaths attributable to meticillin-resistance for the study population in the study period irrespective of the screening regime. In comparison, TB screening may have reduced the number of people with active TB disease, as well as avoiding one death.

Comparing the morbidity and mortality of MRSA and TB can be difficult, as both are diseases that patients can die with but not necessarily of. While the disease progression and death from TB is relatively predictable, MRSA can cause a multitude of different infections of varying severity. There is, however, concern for public health when MRSA is transmitted from person to person in a healthcare setting, rather than in the community via colonisation of healthy individuals. We modelled transmission of MRSA in a healthcare setting and our mortality estimate is derived from the probability of death from severe MRSA infections among inpatients.

The estimates of progression to disease and eventual death of TB in our model accounts for TB cases that would have been diagnosed at a later time, without an effective screening programme. Our screening sensitivity is adjusted downwards from the mean sensitivity found in the literature on laboratory methods, to give a more conservative estimate reflecting that both testing procedures and laboratory methods affect the sensitivity of screening. We also attempted to separate the additional risk associated with MRSA, as compared with MSSA. In most iterations of our simulation, this added risk was small, which is why we were unable to ascertain any attributable risk of mortality to the antibiotic resistance in the bacteria.

A weakness of our model is that it is static and untimed so it can only estimate lifetime probabilities without recovery or recurring infections. The advantage of such a risk assessment model is that the framework is relatively simple and easy to interpret, ideal for risk analyses where rapid choices need to be made between different screening priorities.

Combining the MRSA screening programme and a TB screening programme is difficult and this is a limitation of the model. It is generally considered unethical to perform medical tests on a patient without any medical indications [29]. MRSA rarely causes severe infections in otherwise healthy people [30] and someone can be MRSA positive with no ill effects during their lifetime. Therefore, a positive screening for MRSA among asylum seekers could lead to unnecessary anxiety, especially if they return to their home country with no medical follow-up [31].

If MRSA-positive patients are found at outpatient diagnostic units (where TB tests are performed), it could result in a beneficial increase in infection control measures and support the decision to test for MRSA before TB in some municipalities. However, the spread of MRSA can be contained by standard precautions such as hand hygiene [13]. If the main concern is the spread of MRSA in the Norwegian community (leading to the aforementioned decision), then standard precautions could be extended to the TB diagnostic station to prevent spread of MRSA and preclude the need to test for it before TB.

Our estimated notification rate of MRSA was lower than other European-based studies looking at asylum seekers during the same time period [32,33]. Most of these studies were performed after the asylum seekers had been in the country for a while, whereas we looked at the occurrence of MRSA in asylum seekers within a month of their entry into Norway. Piso et al. [33] conducted a cross-sectional study and found evidence of outbreaks among refugees in the asylum centres, with large variation between and within centres.

We found prevalence of TB in newly arrived asylum seekers similar to what has been reported in other studies [34]. We also found that although the prevalence of TB was higher among the asylum seekers than in their countries of origin, the distribution between countries was relatively consistent [8,35]. Some cases of TB may have been missed as no screening program is 100% effective.

The estimates of the disease occurrence are based on Norwegian register data. While this data is generally of high quality and complete, there are uncertainties regarding the model inputs that may affect the outcomes, such as the estimated screening sensitivity or the reproduction numbers. Both have a major impact on the results of our model, but empirical estimates are hard to find. Nevertheless, this framework was successful in comparing different screening strategies in an emergency scenario and it could be applied to similar situations e.g. possible need to screen for resistant intestinal bacteria.

### Conclusion

The number of newly-arrived asylum seekers in 2015 posed a challenge for healthcare systems all over Europe. In such situations, prioritisations sometimes have to be made between competing interventions. Here, we suggest a model for helping to determine which priorities could be made. Based on the results of model simulations, we conclude that it was reasonable to advise against screening for MRSA before screening for TB among newly arrived asylum seekers in Norway, since such a strategy hindered quick clarification of the asylum seekers’ TB status. The methods used to evaluate our prioritisation and our model can act as a framework to guide others in future and similar situations.

## Supplementary Data

767000 Supplement S1
