# Supplementary material for: Targeting TB or MRSA in Norwegian municipalities during ‘the refugee crisis’ of 2015: a framework for priority setting in screening
Source: Euro Surveill. 2019 Sep 19;24(38):1800676. doi: 10.2807/1560-7917.ES.2019.24.38.1800676 (PMC6761574; doi:10.2807/1560-7917.ES.2019.24.38.1800676)
Supplement: Supplement S1 [file 1800676_DANIELSEN_SupplementS1.pdf]

Supplement S1

Targeting TB or MRSA in Norwegian municipalities during ‘the refugee crisis’ of 2015: a framework for priority setting in screening

Danielsen AS, Elstrøm P, Arnesen TM, Gopinathan U & Kacelnik O

Disclaimer

This supplementary material is hosted by Eurosurveillance as supporting information alongside the article “Targeting TB or MRSA in Norwegian municipalities during ‘the refugee crisis’ of 2015: a framework for priority setting in screening” on behalf of the authors who remain responsible for the accuracy and appropriateness of the content. The same standards for ethics, copyright, attributions and permissions as for the article apply. Supplements are not edited by Eurosurveillance and the journal is not responsible for the maintenance of any links or email addresses provided therein.

TABLE S1. Model equations for clinical outcomes of TB and MRSA in different screening regimes, Norway, 2014–2016

Notation	Clinical outcome	Equation
Y_1	MRSA secondary cases (carriage or colonisation)	$N\alpha\beta(1-\delta)$
Y_2	MRSA infections	$\gamma(Y_1 + N\alpha)$
Y_3	MRSA mortality	$\delta\tau\varepsilon(Y_1 + N\alpha)$
Y_4	MRSA mortality attributable to resistance	$(\delta\tau\varepsilon(Y_1 + N\alpha))-$ $(\delta\tau\,\zeta\,(Y_1 + N\alpha))$
Y_5	Secondary LTBI	$\theta N\eta$
Y_6	Total TB disease	λY_5
Y_7	Total TB mortality	$\mu(N\eta + Y_6)$

LTBI: latent tuberculosis infection; MRSA: meticillin-resistant *S. aureus*; TB: tuberculosis.

FIGURE S1. Sensitivity analysis for the secondary cases outcome for MRSA

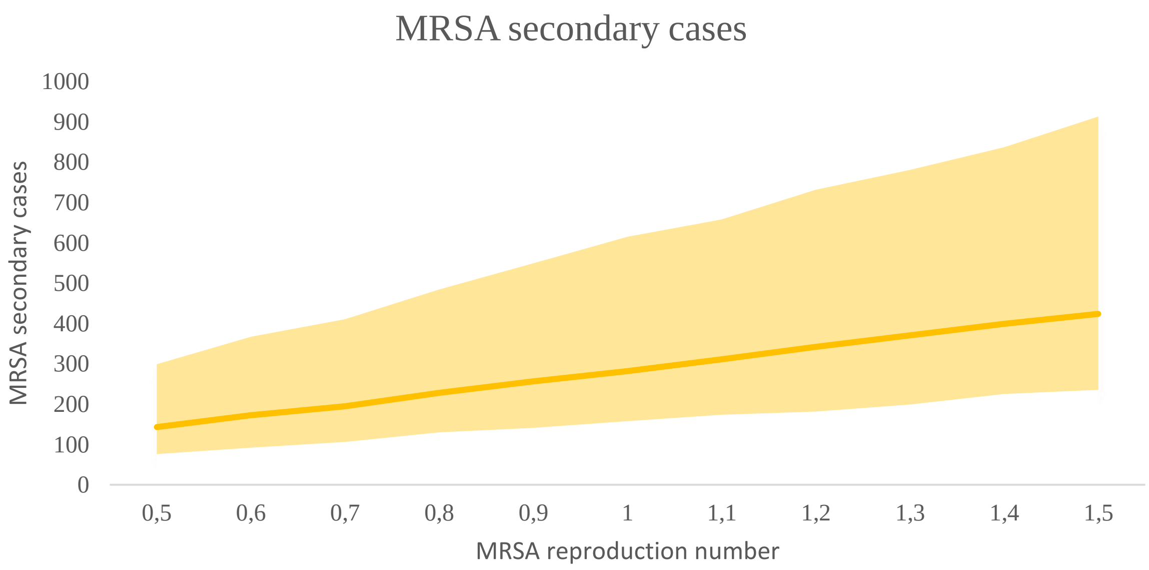

MRSA: meticillin-resistant *Staphylococcus aureus*.

FIGURE 4. Sensitivity analysis for the mortality outcome for meticillin-resistant *S. aureus*

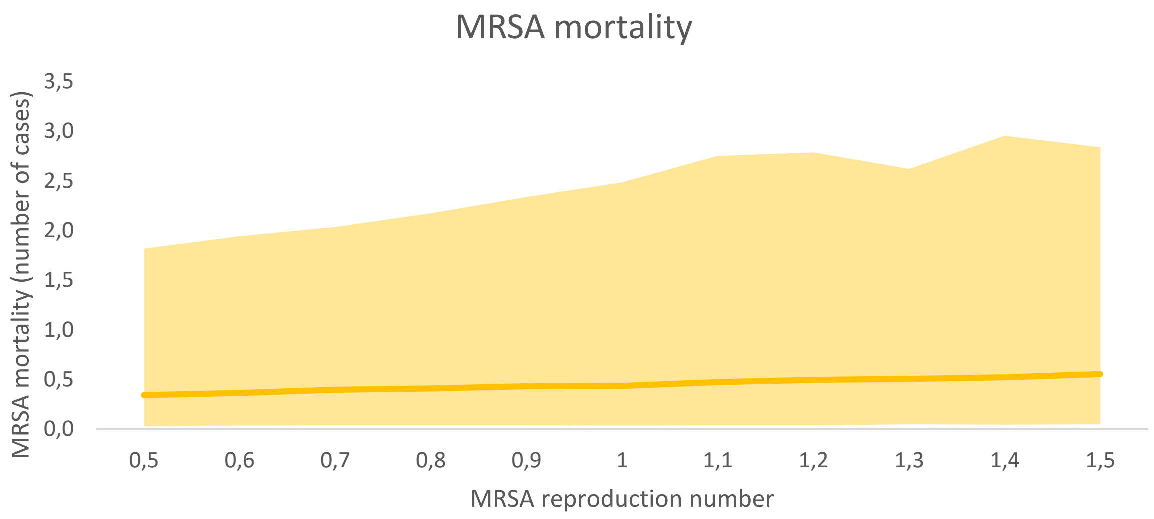

FIGURE 5. Sensitivity analysis for the secondary cases outcome for tuberculosis

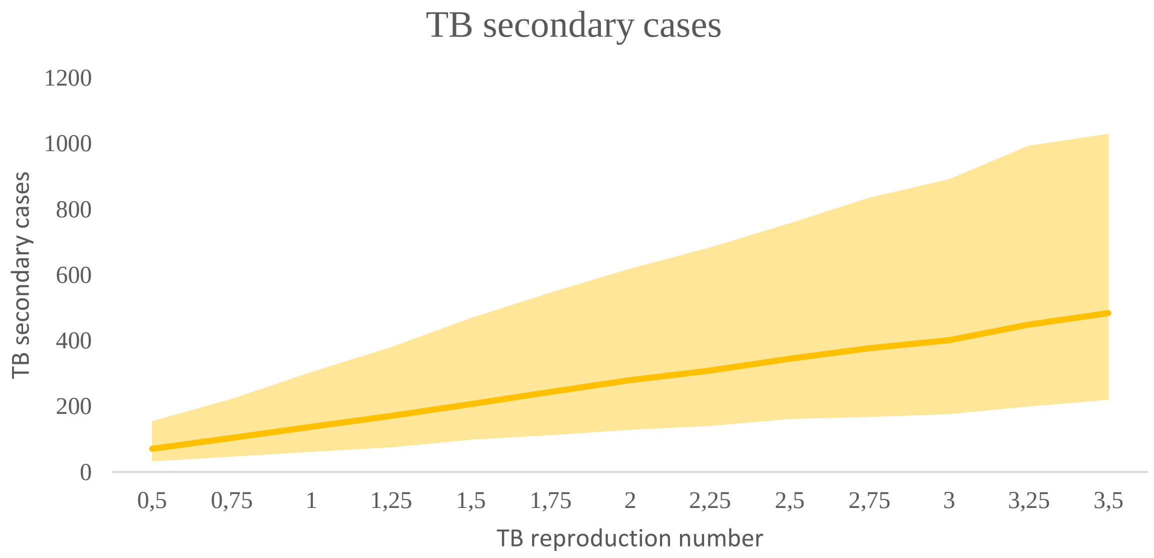

FIGURE 6. Sensitivity analysis for the mortality outcome for tuberculosis

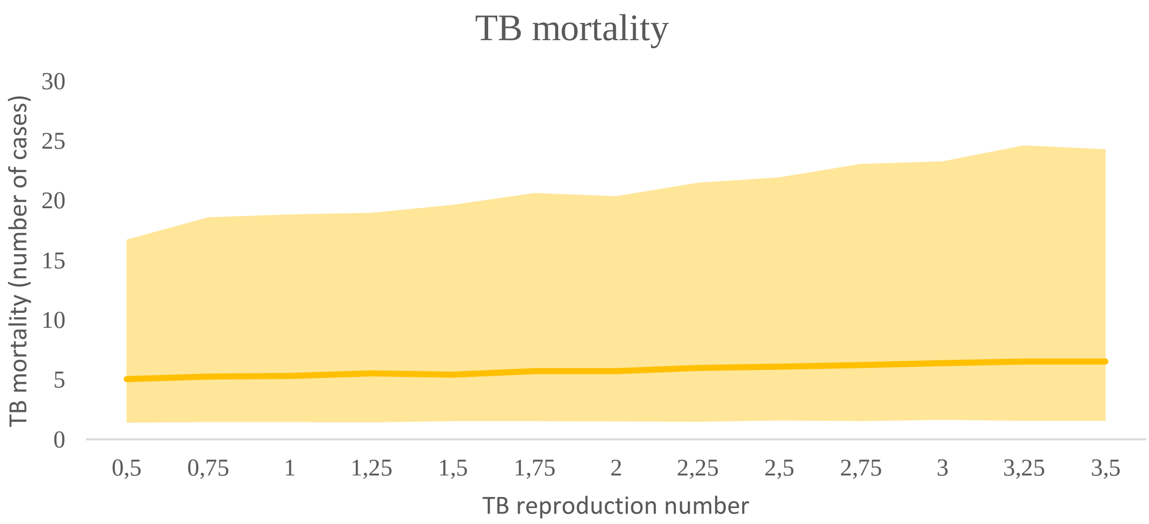
TB mortality
TB mortality (number of cases)
30
25
20
15
10
5
0
0,5
0,75
1
1,25
1,5
1,75
2
2,25
2,5
2,75
3
3,25
3,5
TB reproduction number
